# Effects of risedronate, alendronate, and minodronate alone or in combination with eldecalcitol on bone mineral density, quality, and strength in ovariectomized rats

**DOI:** 10.1016/j.bonr.2021.101061

**Published:** 2021-04-06

**Authors:** Tetsuo Yano, Teppei Ito, Yuya Kanehira, Mei Yamada, Hiromi Kimura-Suda, Hirotaka Wagatsuma, Daisuke Inoue

**Affiliations:** a Research Center, Ajinomoto Pharmaceuticals Co., Ltd., 1-1 Suzuki-cho, Kawasaki-ku, Kawasaki-shi, Kanagawa 210-8681, Japan; b Graduate School of Photonics Science, Chitose Institute of Science and Technology, 758-65 Bibi, Chitose, Hokkaido 066-8655, Japan; c Third Department of Medicine, Teikyo University School of Medicine, 3426-3 Anegasaski, Ichihara-shi, Chiba 299-0111, Japan

**Keywords:** ALF, alfacalcidol, ALN, alendronate, BMD, bone mineral density, BPs, bisphosphonates, ELD, eldecalcitol, FTIR, Fourier transform infrared, micro-CT, micro-computed tomography, MIN, minodronate, OVX, ovariectomized, RIS, risedronate, Risedronate, Alendronate, Minodronate, Eldecalcitol, FTIR imaging, Combination therapy

## Abstract

Combination therapy of active vitamin D_3_ with some bisphosphonates (BPs) has been reported to be clinically beneficial. However, combination therapy of eldecalcitol (ELD) with BP has to date not been validated as to whether it is beneficial in the clinical setting. Preclinical studies suggested that simultaneous treatment with ELD and some BPs is more effective than monotherapy. However, the relative potency of various BPs, when used in combination with ELD, is completely unknown. In this study, we examined and compared the effects of risedronate (RIS), alendronate (ALN), and minodronate (MIN) alone or in combination with ELD on bone mass, microarchitecture, strength, and material properties in ovariectomized Sprague-Dawley rats aged 13 weeks. RIS, ALN, MIN, and ELD were administered five times weekly for 16 weeks. Micro-computed tomography analysis, compression test, and Fourier transform infrared (FTIR) imaging analysis were performed 16 weeks after treatment initiation. Trabecular and cortical bone mineral density (BMD) in the fourth lumbar vertebra (L4) significantly increased in the RIS + ELD, ALN + ELD, and MIN + ELD groups compared with the vehicle group. Moreover, the bone microarchitecture of L4 in all the BP + ELD groups also significantly improved. On mechanical testing of L4, the maximum load was significantly increased in the RIS + ELD and ALN + ELD groups. FTIR analysis revealed that the mineral-to-collagen ratio of trabecular bone in L3 of all the BP + ELD groups was significantly increased compared with the vehicle group. By contrast, the carbonate-to-phosphate ratio, a parameter of mineral immaturity, was significantly decreased in the RIS + ELD and ALN + ELD groups. BP + ELD improved the BMD and structural properties of the bone to a similar extent. RIS + ELD and ALN + ELD also improved bone strength. Furthermore, treatment with BP + ELD improved the bone material. These results suggest that the combination therapy of BP and ELD is beneficial and warrants further clinical trials.

## Highlights

• Combination of bisphosphonates and eldecalcitol was superior to either monotherapy.
• Bone quality on FTIR imaging analysis correlated with bone strength.
• Combined bisphosphonate and eldecalcitol may be useful in the clinical setting.

## 1 Introduction

Nitrogen-containing bisphosphonates (BPs) have been used as first-line drugs to treat osteoporosis for over 30 years (Cosman et al., 2014; Schweser and Crist, 2017). The BPs alendronate (ALN), risedronate (RIS), and minodronate (MIN) have a similar mechanism of anti-resorptive action. They inhibit farnesyl pyrophosphate synthase (FPPS) in the intracellular mevalonate pathway to disrupt protein isoprenylation, thereby causing osteoclast dysfunction and apoptosis (Rogers et al., 2011). The potential for inhibiting the activity of FPPS among these three BPs has been reported as MIN > RIS > ALN (Dunford et al., 2001). Differences in the affinity for hydroxyapatite have also been reported (Rogers et al., 2011). Although such differences between these BPs result in distinct anti-resorptive activity in vitro, this does not necessarily predict clinical efficacy in cases of fracture. RIS has previously been reported to display a significantly greater potency for fracture prevention in patients with osteoporosis than ALN in the first year of therapy (Silverman et al., 2007). We also reported that RIS might exhibit better effects on the bone quality compared with ALN in rat models (Yano et al., 2014; Yano et al., 2017). However, the mechanisms of the distinct effects of each BP remain to be fully understood.

Active vitamin D_3_ drugs, including calcitriol and alfacalcidol (ALF), have been used as therapeutic agents for osteoporosis (Cianferotti et al., 2015). In addition to enhancing the physiological action of vitamin D, thereby increasing the calcium absorption from the small intestine, these drugs have been suggested to regulate bone metabolism through direct action on the bone cells as well as suppression of parathyroid hormone secretion from the parathyroid glands (Ryan et al., 2013; Urena-Torres and Souberbielle, 2014). Nevertheless, the anti-fracture efficacy of active vitamin D_3_ as a monotherapy appears limited (Papadimitropoulos et al., 2002; O'Donnell et al., 2008). Eldecalcitol (ELD), a calcitriol analog developed in Japan, has shown a superior effect to ALF in suppressing bone turnover, increasing the bone mineral density (BMD), and preventing vertebral fracture (Matsumoto and Kubodera, 2007; Matsumoto et al., 2011; Nakamura et al., 2013; Xu et al., 2016), presumably because of its unique pharmacological properties, including higher affinity for vitamin D-binding protein (Okano et al., 1991). ELD has also been reported to increase the bone mass by mini-modeling (Saito et al., 2013) and/or bone formation (Saito et al., 2015).

Several studies have suggested that the addition of active vitamin D_3_ to BP therapy is further beneficial (Ringe et al., 2007; Felsenberg et al., 2011; Orimo et al., 2011; Schacht and Ringe, 2011). Because ELD has a unique anti-resorptive effect in addition to its ability to promote calcium absorption similar to that of authentic active vitamin D_3_, its combination with BPs appears to be a promising treatment. Indeed, some combinations of ELD and BP have already been shown to be clinically more effective in suppressing bone turnover and improving the gain in BMD than monotherapy with BP alone (Sakai et al., 2015a; Ebina et al., 2016), although the effect on the fracture incidence has not been reported. An animal study demonstrated that ALN in combination with ELD significantly improved the BMD and bone strength compared with ALN monotherapy in ovariectomized (OVX) rats (Sakai et al., 2012). Another study in rats similarly indicated that a combination of ALN and ELD was superior in increasing the bone mass and improving the quality compared with a combination of ALN and ALF (Sugimoto et al., 2013). These studies also suggested that ALN and ELD improved bone microarchitecture by different mechanisms. Considering the unique characteristics of each BP, it is plausible that different BPs might display distinct effects on bone mass and quality when combined with ELD.

In the present study, we examined the effects of three BPs alone or in combination with ELD in OVX rats. To determine the drug effects on bone quality, we performed Fourier transform infrared (FTIR) spectroscopic imaging in addition to microstructural analysis by micro-computed tomography (micro-CT). FTIR imaging is a powerful tool to assess bone quality in that it can determine the mineral-to-collagen matrix (PO_4_^3−^/amide I) and carbonate-to-phosphate (CO_3_^2−^/PO_4_^3−^) ratios, crystallinity, mineral maturity, and collagen cross-links and fiber orientation (Paschalis et al., 2011). However, it has never been applied to evaluate the effect of active vitamin D_3_ or its analog, except for native vitamin D_3_ supplementation (Paschalis et al., 2017). This study is the first direct comparison of the effects of three different BPs in combination with ELD, including quality analysis by FTIR imaging.

## 2 Materials and methods

### 2.1 Reagents and animals

RIS was synthesized by Takeda Pharmaceutical Company (Tokyo, Japan). ALN, MIN, and ELD were purchased from Wako Pure Chemical Industries (Tokyo, Japan), Chengdu-D-Innovation pharmaceutical (Chengdu, China), and Chugai Pharmaceutical (eldecalcitol capsule, Tokyo, Japan), respectively. Saline and medium-chain triglyceride oil (MCT-oil) were obtained from Otsuka Pharmaceutical (Tokushima, Japan) and Nisshin Oillio Group, Ltd. (Tokyo, Japan), respectively. Isoflurane was purchased from Mylan (Tokyo, Japan). Eighty-one female Sprague-Dawley rats aged 12 weeks were purchased from Charles River Laboratories Japan (Yokohama, Japan). The animals were housed three per cage in a room with a light/dark cycle of 12/12 h and were acclimated to the study conditions for 1 week prior to the start of the experiment. All animals were paired-fed rodent food (CRF-1 formula, Charles River Laboratories Japan). Water from Ajinomoto Pharmaceuticals in-house production was available ad libitum. The experimental protocol was approved by the Animal Care and Use Committee of Ajinomoto Pharmaceuticals Co., Ltd., which was operated in accordance with the Guideline for Proper Conduct of Animal Experiments (Science Council of Japan, 2006).

### 2.2 Experimental design

The animals when aged 13 weeks were assigned to sham surgery (sham, *n* = 9) or OVX (*n* = 72) groups by matching their body weight as well as the trabecular BMD of the fourth lumbar vertebra (L4) and the cortical BMD of the left femur by micro-computed tomography (micro-CT, Skyscan1176; Bruker microCT, Kontich, Belgium) using SAS 9.3 (SAS Institute, Cary, USA). The OVX group was further subdivided into the following eight groups: (1) vehicle (saline and MCT-oil), (2) RIS, (3) ALN, (4) MIN, (5) ELD, (6) RIS + ELD, (7) ALN + ELD, and (8) MIN + ELD, with nine animals in each group. RIS (0.7 μg/kg), ALN (1.4 μg/kg), and MIN (0.14 μg/kg) were injected subcutaneously five times weekly for 16 weeks. ELD (0.04 μg/kg) was administered orally five times weekly for 16 weeks. Saline and MCT-oil were administered subcutaneously and orally, respectively, five times weekly for 16 weeks in the vehicle and sham groups. Drug administration was initiated on the day after OVX or sham surgery. The animals were euthanized by exsanguination under anesthesia with isoflurane (2.5–3%) at 16 weeks after the initiation of drug administration and the lumbar vertebrae and femurs were evaluated for the BMD and microarchitecture by micro-CT, bone strength by mechanical testing, and bone quality by FTIR imaging analysis.

### 2.3 Structural analysis by micro-CT

To investigate the effects of BP alone or in combination with ELD treatment on the trabecular and cortical microarchitecture of the L4 and distal femur, in vivo three-dimensional trabecular and cortical analyses were performed by micro-CT. Animals were anesthetized with isoflurane (2.5–3%) before being scanned in a supine position on an animal bed with the left hind leg fixed in a cylindrical plastic holder. The trabecular bone of the femur and the trabecular and cortical bone of the lumbar vertebrae were scanned in 50 slices (0.19 cm total thickness). The cortical bone of the femur was scanned in 130 slices (0.5 cm total thickness). In the trabecular bone, we determined the following histomorphometric parameters on the resultant three-dimensional images: bone volume fraction (BV/TV, %), trabecular number (Tb.N, 1/mm), trabecular thickness (Tb.Th, mm), trabecular separation (Tb.Sp, mm), connectivity density (1/mm^3^), and BMD (g/cm^3^). In the cortical bone, we measured the following histomorphometric parameters on the two-dimensional images: cortical bone area (Ct.Ar, mm^2^), cortical thickness (Ct.Th, mm), and BMD (g/cm^3^) (Dempster et al., 2013). The analysis was performed using CTAn (ver. 1.11.4.2, Bruker).

### 2.4 Mechanical testing

The fourth lumbar vertebrae (L4) were tested for bone strength by a compression test using a previously described method with slight modification (Ohnishi et al., 1997). The left femurs were tested for bone strength using the three-point bending test (Yano et al., 2014). Biomechanical testing was performed using the TK-252/MK (Muromachi Kikai, Tokyo, Japan). For compression of the vertebrae, the L4 was fixed on a holder with an adhesive agent and placed on the testing platform. In three-point bending of the femur, the two support points below the bone were interspaced at 13 mm. The deformation rates of the vertebrae and femurs were 5 and 2 mm/min, respectively. From the load-deformation curve, the stiffness (N/mm) was calculated as previously reported (Turner and Burr, 1993). The maximum load (N) was obtained directly from the load-deformation curve. Stiffness was defined by the slope of the ascending portion of the load-deformation curve between 30% and 70% of the maximum load value.

### 2.5 FTIR imaging analysis

The right femurs and third lumbar vertebrae (L3) were removed and embedded in polymethyl methacrylate (PMMA). Longitudinal sections of 3-μm thickness were prepared using a microtome (Aparicio et al., 2002). FTIR images of the longitudinal sections were acquired using an FTIR imaging system with a mercury‑cadmium-telluride linear array detector (Spotlight 400 system, PerkinElmer, Waltham, MA, USA) in transmittance mode with a frequency range of 4000–680 cm^−1^, a resolution of 4 cm^−1^, and a pixel size of 25 × 25 μm. The background spectrum was obtained through a barium fluoride window. Thirty FTIR spectra extracted from each trabecular and cortical bone in the image were obtained with baseline collection and PMMA spectral subtraction using Spectrum 10 software (PerkinElmer) and subsequently used to evaluate the PO_4_^3−^/amide I and CO_3_^2−^/PO_4_^3−^ in the femur and L3. The PO_4_^3−^/amide I was calculated by integrating the area of the phosphate band (PO_4_^3−^, 1181–906 cm^−1^) and dividing it by the area of the amide I band (1712–1609 cm^−1^). The CO_3_^2−^/PO_4_^3−^ was calculated by integrating the area of the carbonate band (CO_3_^2−^, 890–851 cm^−1^) and dividing it by the area of the PO_4_^3−^ band.

### 2.6 Statistical analysis

All data are expressed as mean ± standard deviation (SD). According to the results of the Shapiro–Wilk test for normal distribution, group differences were analyzed by *t*-test or Wilcoxon test and analysis of variance with post-hoc Dunnett's test, Tukey test, Steel or Steel–Dwass test on JMP (ver. 12.0.1; SAS Institute, Tokyo, Japan). *P* values of <0.05 were considered statistically significant.

## 3 Results

### 3.1 BMD, bone microarchitecture, and mechanical testing of the fourth lumbar vertebrae

**Fig. 1:**
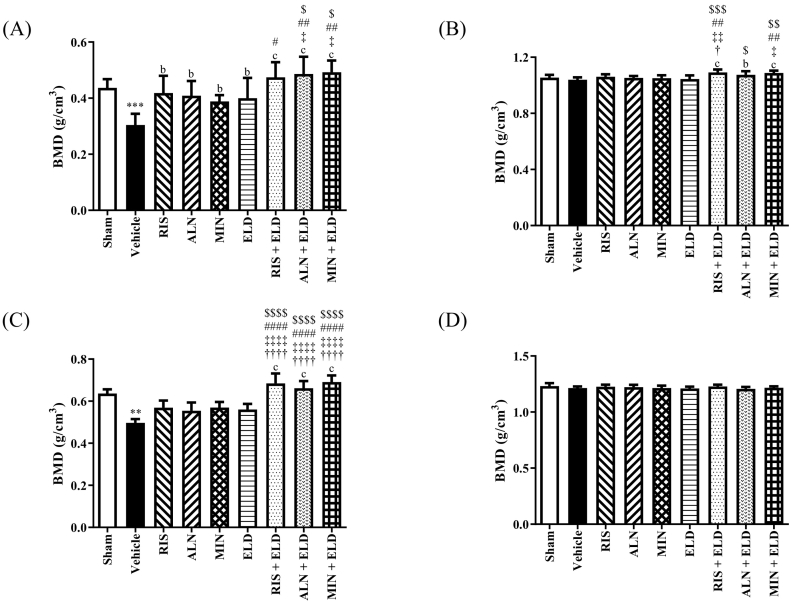
Effect of bisphosphonates, eldecalcitol, and their combinations on the L4 and femoral BMD determined by micro-CT. Lumber vertebral bones (L4) and left femurs were harvested from sham and OVX rats after 16 weeks. (A) Trabecular bone of L4, (B) cortical bone of L4, (C) femoral trabecular bone, and (D) femoral cortical bone. Data are presented as mean + SD. *n* = 9. ^⁎⁎^*p* < 0.01 and ^⁎⁎⁎^*p* < 0.001 compared with the sham group. ^b^*p* < 0.01 and ^c^*p* < 0.001 compared with the vehicle group. ^†^*p* < 0.05 and ^††††^*p* < 0.0001 compared with RIS. ^‡^*p* < 0.05, ^‡‡^*p* < 0.01, and ^‡‡‡‡^*p* < 0.0001 compared with ALN. ^#^*p* < 0.05, ^##^*p* < 0.01, and ^####^*p* < 0.0001 compared with MIN. ^$^*p* < 0.05, ^$$^*p* < 0.01, ^$$$^*p* < 0.001, and ^$$$$^*p* < 0.0001 compared with ELD. Abbreviations: BMD, bone mineral density; ALN, alendronate; ELD, eldecalcitol; micro-CT, micro-computed tomography; MIN, minodronate; RIS, risedronate.

Trabecular BMD and bone microarchitecture in the L4 vertebrae were significantly lower in the vehicle-treated OVX rats than in the sham group (Fig. 1A). All the drug groups displayed a significantly increased trabecular BMD compared with the vehicle group. ALN + ELD and MIN + ELD significantly increased the trabecular BMD compared with that of each monotherapy (Fig. 1A). All combinations of BP + ELD and almost all the monotherapies consistently and significantly improved the trabecular bone microarchitecture compared with that in the vehicle group (Table 1). In particular, the Tb.N in the ALN + ELD and MIN + ELD groups was higher compared with each BP alone, and all combinations with BP were better than ELD alone.

**Table 1:** Effects of BP, ELD, or combinations of BP and ELD on the L4 microarchitecture in OVX rats.

Parameter	Sham	Vehicle	RIS	ALN	MIN	ELD	RIS + ELD	ALN + ELD	MIN + ELD
Trabecular bone									
BV/TV (%)	32.18 ± 3.60	18.96 ± 4.00***	29.88 ± 6.96b	28.54 ± 5.64b	26.30 ± 2.54	28.45 ± 8.09b	36.29 ± 6.51c, #	38.00 ± 6.98c, ‡, ##, $	38.45 ± 4.41c, ‡, #, $
Tb.Th (mm)	0.12 ± 0.01	0.11 ± 0.01**	0.12 ± 0.01	0.12 ± 0.01	0.11 ± 0.01	0.12 ± 0.01a	0.13 ± 0.01b	0.13 ± 0.01b	0.13 ± 0.01c, #
Tb.N (1/mm)	2.60 ± 0.18	1.69 ± 0.32***	2.52 ± 0.39c	2.39 ± 0.36c	2.31 ± 0.16b	2.27 ± 0.49b	2.85 ± 0.26c, #, $$	2.96 ± 0.28c, ‡‡, ##, $$$	2.97 ± 0.27c, ‡‡, ##, $$$
Tb.Sp (mm)	0.26 ± 0.02	0.34 ± 0.03***	0.25 ± 0.03c	0.26 ± 0.04c	0.26 ± 0.02c	0.29 ± 0.05b	0.24 ± 0.02c, $$	0.22 ± 0.02c, $$$	0.23 ± 0.03c, $$
Conn.D (1/mm^3^)	58.65 ± 10.87	36.74 ± 10.15***	63.51 ± 15.6c	59.89 ± 9.04c	58.25 ± 9.57b	48.94 ± 14.27	64.41 ± 8.48c	72.21 ± 6.78c, $$	67.70 ± 15.39c, $
Cortical bone									
Ct.Ar (mm^2^)	3.18 ± 0.23	2.87 ± 0.26*	3.18 ± 0.38	3.07 ± 0.34	3.15 ± 0.16	3.22 ± 0.45	3.92 ± 0.40c, ††, ‡‡‡, ##, $$	4.10 ± 0.54c, †††, ‡‡‡‡, ####, $$$	3.97 ± 0.36c, ††, ‡‡‡, ###, $$
Ct.Th (mm)	0.18 ± 0.01	0.17 ± 0.01	0.18 ± 0.01	0.17 ± 0.01	0.18 ± 0.01	0.18 ± 0.02	0.21 ± 0.02c, ††, ‡‡‡, ##, $$$	0.21 ± 0.02c, ††, ‡‡‡, ##, $$	0.21 ± 0.02c, †††, ‡‡‡‡, ###, $$$$

Data are presented as mean ± SD. n = 9. ^⁎^*p* < 0.05 and ^⁎⁎^*p* < 0.01 compared with the sham group. ^a^*p* < 0.05, ^b^*p* < 0.01, and ^c^*p* < 0.001 compared with the vehicle group. ^†^*p* < 0.05, ^††^*p* < 0.01, and ^†††^*p* < 0.01 compared with RIS. ^‡^*p* < 0.05, ^‡‡^*p* < 0.01, ^‡‡‡^*p* < 0.001, and ^‡‡‡‡^*p* < 0.0001 compared with ALN. ^#^*p* < 0.05, ^##^*p* < 0.01, ^###^*p* < 0.001, and ^####^*p* < 0.0001 compared with MIN. ^$^*p* < 0.05, ^$$^*p* < 0.01, ^$$$^*p* < 0.001, and ^$$$$^*p* < 0.0001 compared with ELD. Abbreviations: ALN, alendronate; BP, bisphosphonate; Conn D, connectivity density; ELD, eldecalcitol; L4, fourth lumbar vertebra; MIN, minodronate; OVX, ovariectomized; RIS, risedronate.

All combinations significantly increased the cortical BMD in L4 compared with that in the vehicle group, whereas the BMD changes by each monotherapy were non-significant (Fig. 1B). Additionally, RIS + ELD and MIN + ELD significantly increased the cortical BMD compared with that of each monotherapy (Fig. 1B). The Ct.Ar was significantly decreased in the vehicle-treated OVX rats compared with that in the sham group. All combination therapies significantly increased the Ct.Ar and Ct.Th compared with that in the vehicle group and in each monotherapy group (Table 1).

**Fig. 2:**
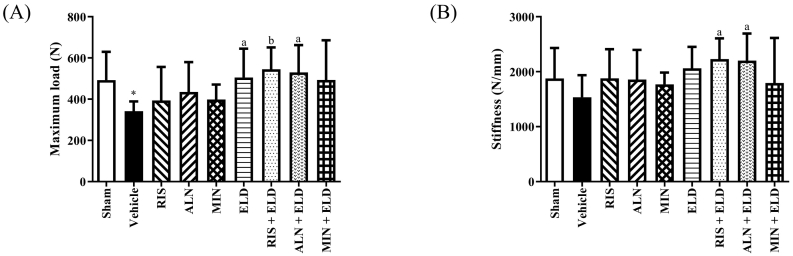
Effect of bisphosphonates, eldecalcitol, and their combinations on the L4 bone strength in OVX rats. Lumber vertebral bones (L4) were harvested from sham and OVX rats after 16 weeks. (A) Maximum load and (B) stiffness. Data are presented as mean + SD. n = 9. ^⁎^*p* < 0.05 compared with the sham group. ^a^*p* < 0.05 and ^b^*p* < 0.01 compared with the vehicle group. Abbreviations: ALN, alendronate; ELD, eldecalcitol; MIN, minodronate; OVX, ovariectomized; RIS, risedronate.

The compression test for L4 bone strength revealed that the maximum load in the vehicle-treated OVX rats was significantly lower than in the sham group (Fig. 2A). Stiffness was also slightly decreased in the vehicle group without statistical significance (Fig. 2B). RIS + ELD and ALN + ELD significantly increased the maximum load and stiffness compared with that in the vehicle group. MIN + ELD also increased these parameters (+44% and + 17%, respectively); however, the effect did not reach statistical significance (Fig. 2A and B). None of the BPs significantly improved the mechanical parameters when used alone. Therefore, RIS and ALN improved mechanical properties of the vertebral body when used in combination with ELD.

### 3.2 BMD and bone microarchitecture of the distal femur

In the analysis of femurs, the vehicle group displayed significantly reduced trabecular BMD and deteriorated bone microarchitecture of the distal femur compared with the sham group (Fig. 1C and Table 2). Combination therapy significantly increased the trabecular BMD of the distal femur compared with that in the vehicle group and each monotherapy group (Fig. 1C). Additionally, combination therapy significantly improved the BV/TV, Tb.Th, Tb.N, and Tb.Sp compared with that in the vehicle group (Table 2). Moreover, combination therapy significantly improved the BV/TV, Tb.Th, and Tb.Sp compared with that of each monotherapy, clearly demonstrating the beneficial effects of co-treatment with BP and ELD. None of the drug-treated groups displayed significant changes in the BMD (Fig. 1D), bone microarchitecture (Table 2), or strength (data not shown) in the cortical bone of the distal femur at 16 weeks.

**Table 2:** Effects of BP, ELD, or combinations of BP and ELD on the femoral microarchitecture in OVX rats.

Parameter	Sham	Vehicle	RIS	ALN	MIN	ELD	RIS + ELD	ALN + ELD	MIN + ELD
Trabecular bone									
BV/TV (%)	56.47 ± 6.65	19.01 ± 4.00***	35.57 ± 10.79	32.27 ± 11.47	35.98 ± 8.35	34.33 ± 7.36	71.33 ± 13.62c, ††††, ‡‡‡‡, ####, $$$$	65.77 ± 10.78c, ††††, ‡‡‡‡, ####, $$$$	72.30 ± 8.14c, ††††, ‡‡‡‡, ####, $$$$
Tb.Th (mm)	0.19 ± 0.02	0.15 ± 0.00***	0.16 ± 0.02	0.16 ± 0.02	0.17 ± 0.01	0.17 ± 0.01	0.28 ± 0.06c, ††††, ‡‡‡‡, ####, $$$$	0.24 ± 0.03c, †††, ‡‡‡, ###, $$$	0.29 ± 0.04c, ††††, ‡‡‡‡, ####, $$$$,¶
Tb.N (1/mm)	2.94 ± 0.20	1.25 ± 0.24***	2.14 ± 0.40	1.96 ± 0.49	2.16 ± 0.37a	2.04 ± 0.31	2.60 ± 0.17c, ‡‡, $$	2.76 ± 0.15c, ††, ‡‡‡‡, ##, $$$	2.54 ± 0.21c, ‡‡, $
Tb.Sp (mm)	0.21 ± 0.02	0.69 ± 0.12***	0.32 ± 0.08a	0.41 ± 0.12	0.31 ± 0.07a	0.42 ± 0.08#	0.18 ± 0.03c, ††, ‡‡‡‡, ##, $$$$	0.20 ± 0.03c, †, ‡‡‡‡, #, $$$$	0.19 ± 0.03c, ††, ‡‡‡‡, #, $$$$
Conn.D (1/mm^3^)	71.88 ± 7.55	33.81 ± 4.88***	84.80 ± 12.06c	70.29 ± 12.00c	80.91 ± 9.99c	51.64 ± 3.82††††, ‡, ####	60.22 ± 18.88a, †††, ##	66.70 ± 11.34b, †	53.46 ± 9.05††††, ###
Cortical bone									
Ct.Ar (mm2)	5.92 ± 0.41	6.29 ± 0.32*	6.24 ± 0.39	6.13 ± 0.28	6.39 ± 0.29	6.64 ± 0.34‡	6.61 ± 0.35	6.38 ± 0.30	6.58 ± 0.42
Ct.Th (mm)	0.62 ± 0.02	0.63 ± 0.03	0.64 ± 0.02	0.62 ± 0.02	0.64 ± 0.03	0.66 ± 0.02‡	0.65 ± 0.02	0.63 ± 0.03	0.65 ± 0.03

Data are presented as mean ± SD. n = 9. ^⁎^*p* < 0.05, ^⁎⁎^*p* < 0.01, and ^⁎⁎⁎^*p* < 0.001 compared with the sham group. ^a^*p* < 0.05, ^b^*p* < 0.01, and ^c^*p* < 0.001 compared with the vehicle group. ^†^*p* < 0.05, ^††^*p* < 0.01, and ^†††^*p* < 0.001 compared with RIS. ^‡^*p* < 0.05, ^‡‡^*p* < 0.01, ^‡‡‡^*p* < 0.001, and ^‡‡‡‡^*p* < 0.0001 compared with ALN. ^#^*p* < 0.05, ^##^*p* < 0.01, ^###^*p* < 0.001, and ^####^*p* < 0.0001 compared with MIN. ^$^*p* < 0.05, ^$$^*p* < 0.01, ^$$$^*p* < 0.001, and ^$$$$^*p* < 0.0001 compared with ELD. ^¶^*p* < 0.05 compared with ALN + ELD. Abbreviations: ALN, alendronate; BP, bisphosphonate; Conn D, connectivity density; ELD, eldecalcitol; MIN, minodronate; OVX, ovariectomized; RIS, risedronate.

### 3.3 FTIR imaging analysis

**Fig. 3:**
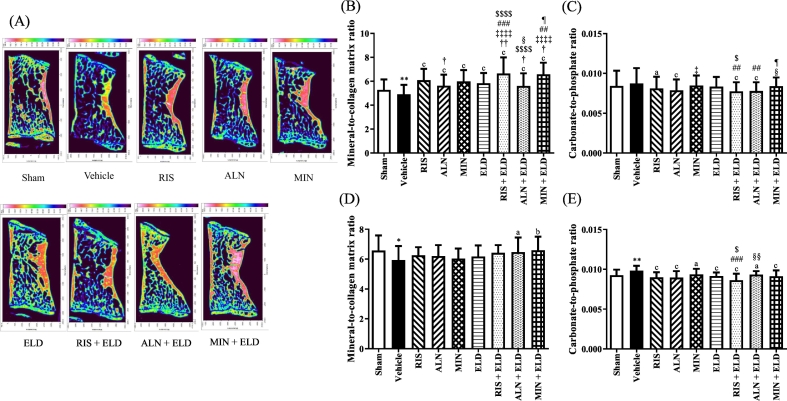
FTIR analysis and bone quality of L3 in OVX rats treated with bisphosphonates, eldecalcitol, or their combinations. Lumber vertebral bones were harvested from sham and OVX rats after 16 weeks. (A) FTIR images with areas of high absorbance for the PO_4_^3−^ band shown as white (or red), and areas of low absorbance for the PO_4_^3−^ band shown as dark blue. (B) Mineral-to-collagen matrix ratio of trabecular bone, (C) carbonate-to-phosphate ratio of trabecular bone, (D) mineral-to-collagen matrix ratio of cortical bone, and (E) carbonate-to-phosphate ratio of cortical bone. Data are presented as mean + SD. *n* = 3 (30 × 3 spectra). ^⁎^*p* < 0.05 and ^⁎⁎^*p* < 0.01 compared with the sham group. ^a^*p* < 0.05, ^b^*p* < 0.01, and ^c^*p* < 0.001 compared with the vehicle group. ^†^*p* < 0.05 and ^††^*p* < 0.01 compared with RIS. ^‡‡‡‡^*p* < 0.0001 compared with ALN. ^##^*p* < 0.01 and ^###^*p* < 0.001 compared with MIN. ^$^*p* < 0.05 and ^$$$$^*p* < 0.0001 compared with ELD. ^§^*p* < 0.05 and ^§§^*p* < 0.01 compared with RIS + ELD. ^¶^*p* < 0.05 compared with ALN + ELD. Abbreviations: ALN, alendronate; ELD, eldecalcitol; FTIR, Fourier transform infrared; MIN, minodronate; OVX, ovariectomized; RIS, risedronate.

**Fig. 4:**
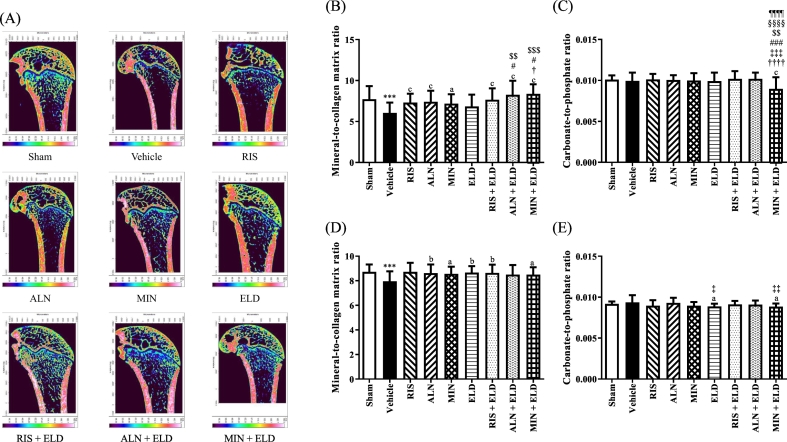
FTIR analysis and bone quality of the femurs in OVX rats treated with bisphosphonates, eldecalcitol, and their combinations. Right femoral bones were harvested from sham and OVX rats after 16 weeks. (A) FTIR images with areas of high absorbance for the PO_4_^3−^ band shown as white (or red), and areas of low absorbance for the PO_4_^3−^ band shown as dark blue. (B) Mineral-to-collagen matrix ratio of trabecular bone, (C) carbonate-to-phosphate ratio of trabecular bone, (D) mineral-to-collagen matrix ratio of cortical bone, and (E) carbonate-to-phosphate ratio of cortical bone. Data are presented as mean + SD. n = 3 (30 × 3 spectra). ^⁎⁎^*p* < 0.001 compared with the sham group. ^a^*p* < 0.05, ^b^*p* < 0.01, and ^c^*p* < 0.001 compared with the vehicle group. ^†^*p* < 0.05 and ^††††^*p* < 0.0001 compared with RIS. ^‡^*p* < 0.05, ^‡‡^*p* < 0.01, and ^‡‡‡^*p* < 0.001 compared with ALN. ^#^*p* < 0.05 and ^###^*p* < 0.001 compared with MIN. ^$$^*p* < 0.01 and ^$$$^*p* < 0.001 compared with ELD. ^§§§§^*p* < 0.0001 compared with RIS + ELD. ^¶¶¶¶^*p* < 0.0001 compared with ALN + ELD. Abbreviations: ALN, alendronate; ELD, eldecalcitol; FTIR, Fourier transform infrared; MIN, minodronate; OVX, ovariectomized; RIS, risedronate.

Fig. 3, Fig. 4 show FTIR images and bone quality-related parameters of the L3 and femurs, respectively. In L3, the vehicle group displayed a significantly decreased PO_4_^3−^/amide I in both trabecular and cortical bone compared with the sham group (Fig. 3B and D). All the drug-treated groups showed a significantly increased PO_4_^3−^/amide I in the trabecular bone compared with the vehicle group (Fig. 3B). Moreover, RIS + ELD and MIN + ELD significantly increased the PO_4_^3−^/amide I compared with that of each monotherapy. The cortical PO_4_^3−^/amide I of L3 was significantly increased in the ALN + ELD and MIN + ELD groups compared with that in the vehicle-treated OVX rats (Fig. 3D).

By contrast, the CO_3_^2−^/PO_4_^3−^ in the L3 trabecular bone tended to increase in the vehicle-treated OVX rats compared with that in the sham group and significantly decreased on treatment with RIS or ALN alone or in combination with ELD (Fig. 3C). The CO_3_^2−^/PO_4_^3−^ of the trabecular bone in the MIN + ELD group was significantly higher compared with the RIS + ELD and ALN + ELD groups (Fig. 3C). Similarly, in the cortical bone in L3 (Fig. 3E), the CO_3_^2−^/PO_4_^3−^ was significantly increased in the vehicle-treated OVX rats compared with that in the sham group, and all drugs caused a significant decrease in the CO_3_^2−^/PO_4_^3−^.

In both trabecular and cortical bone of the femur, the PO_4_^3−^/amide I was significantly decreased in the vehicle-treated OVX rats compared with that in the sham group (Fig. 4B and D). Almost all the drugs significantly increased the PO_4_^3−^/amide I compared with that in the vehicle group (Fig. 4B and D). Treatment with ALN + ELD and MIN + ELD significantly increased the PO_4_^3−^/amide I compared with that with ELD in the trabecular bone (Fig. 4B). In the cortical bone of the femur, almost all the drugs significantly increased the PO_4_^3−^/amide I; however, the benefit of combination therapy was not evident (Fig. 4D).

Regarding the CO_3_^2−^/PO_4_^3−^ in the femur, OVX did not cause any significant changes in either the trabecular or cortical bone (Fig. 4C and E). BP alone and in combination with ELD displayed little or no effect on the CO_3_^2−^/PO_4_^3−^, except MIN + ELD, which significantly decreased the ratio compared with that in the vehicle group (Fig. 4C and E).

## 4 Discussion

In the present study, we investigated the effect of three different BPs, alone and in combination with ELD, by direct comparison in OVX rats. The results indicated that ELD co-treatment with each of the three BPs tested displayed consistent beneficial effects on bone mass and microarchitecture on micro-CT as well as on material properties evaluated by FTIR, and consequently, on mechanical strength.

Regarding the volumetric BMD, BPs and ELD displayed apparent additive or synergistic effects, particularly in L4. The increase in the trabecular and cortical BMD of the lumbar spine appears to be directly reflected in the improved mechanical properties, as demonstrated by the compression test. The advantage of combination therapy with BP and ELD over BP monotherapy is consistent with previous reports based on dual energy X-ray absorptiometry-measured areal BMD using ALN (Sakai et al., 2012; Sugimoto et al., 2013) or ibandronate (Sakai et al., 2015b). In the femur, we observed a prominent positive effect of combination therapy on the trabecular BMD in contrast to little if any effect on the cortical bone. We also observed clear effects on the trabecular structural parameters, including the Tb.Th and Tb.Sp. The results suggested that the previously reported increase in femoral areal BMD (Sakai et al., 2012; Sugimoto et al., 2013; Sakai et al., 2015a) by combination therapy with BP and ELD was mostly attributed to the effect on the trabecular bone.

The current study was the first to evaluate the effect of ELD alone or in combination with BPs on bone quality by FTIR analysis. FTIR imaging analysis of L3 revealed that all the BPs and their combination therapy with ELD significantly improved the PO_4_^3−^/amide I in the trabecular bone, which had been reduced by ovariectomy. Moreover, the improvement in the PO_4_^3−^/amide I was greater with the combination therapy with ELD compared with that of each BP alone. These results indicated that the addition of ELD to BPs further improved the PO_4_^3−^/a (Aparicio et al., 2002) mide I in the trabecular bone of the lumbar vertebrae, which most likely additionally contributed to better mechanical strength. It is notable that changes in the PO_4_^3−^/amide I by combination therapy appeared to be parallel to those in the volumetric BMD; however, the additive effect of ELD was relatively modest. It thus appears likely that co-treatment with ELD induced an increase in the BMD as well as improved microarchitecture, ultimately resulting in greater physical strength of the bone tissue without hypermineralization. Conversely, an increased PO_4_^3−^/amide I also suggested that, at least in the context of combination therapy with BPs, the contribution of mini-modeling-based new bone formation to an increase in the BMD was minor.

All the ELD and BP combinations reversed the ovariectomy-induced increase in the CO_3_^2−^/PO_4_^3−^ in both the trabecular and the cortical bone of L3. Our observations are consistent with previous studies (Hara et al., 2007; Gadeleta et al., 2000) that have shown that ovariectomy increased the CO_3_^2−^/PO_4_^3−^ in the femurs of OVX rats and lumbar vertebrae of OVX monkeys. The increased CO_3_^2−^/PO_4_^3−^ in OVX animals is considered to result from a reduction of mineral maturity. Our results suggested that the mineral in OVX rats was poorly crystallized apatite, and treatment with all the BPs or their combinations with ELD improved the mineral maturity, increasing the content of stoichiometric apatite in both trabecular and cortical bone in L3. Moreover, the effect of RIS + ELD and ALN + ELD on the CO_3_^2−^/PO_4_^3−^ in trabecular bone of L3 correlated with the bone strength. Therefore, bone strength was strongly reflected by mineral maturity in the present study. In the femur, no reduction of the CO_3_^2−^/PO_4_^3−^ by combination therapy was detected, except in rats treated with MIN + ELD. The reason for this result is currently unknown, although the strongest anti-resorptive activity of MIN among the three BPs investigated in the current study (Dunford et al., 2001) might be in some way associated with the large increase in the PO_4_^3−^/amide I and decrease in the CO_3_^2−^/PO_4_^3−^. The significance of this observation remains to be determined. Although crystallinity and collagen cross-links were evaluated in the present study, all BPs and their combination with ELD did not induce significant changes compared with the vehicle group (data not shown). A previous study (Durchschlag et al., 2006) showed that RIS significantly decreased the crystallinity and collagen cross-links near bone-forming sites in trabecular bone of iliac crest biopsies in a time-dependent manner. However, RIS did not affect these parameters near the resorbing sites. This may be because the sites where we analyzed these parameters were randomly chosen from the whole bone of L3 in this study. We speculate that the effect of ELD and BPs on these parameters is significant only when the forming and resorbing sites are analyzed separately after a long-term treatment.

There are limitations to the present study. First, the dosage of each of the three BPs was determined empirically based on the literature and our experience, so that it was proportional to the clinical dosage. Second, because BPs and ELD were administered immediately after OVX, the observed effect was more representative of prevention rather than treatment, and may thus not be applicable to established postmenopausal osteoporosis. The relevance of our findings to the clinical setting should thus be carefully evaluated. Finally, the study was not designed to fully elucidate the mechanism of drug action and did not provide information about bone metabolism using biochemical markers or dynamic histomorphometry. Nonetheless, the strength of the current study is the fact that three BPs were tested at the same time, allowing direct comparison for the first time in the context of co-treatment with ELD. This study is also novel in that the effect of combination therapy with BP and ELD on the material properties of the bone was evaluated by FTIR imaging.

## 5 Conclusion

Combinations of ELD and three nitrogen-containing BPs, namely RIS, ALN, and MIN, significantly improved the bone mass and microarchitecture in OVX rats, consequently leading to increased mechanical strength. The improvement with the combination therapy was significantly superior to that with each BP or ELD alone. FTIR imaging analysis excluded hypermineralization and compromised mineral maturity. Therefore, combination therapy with ELD and BP can be validated as a potential therapeutic approach for the treatment of osteoporosis in the clinical setting.

## Transparency document

Transparency document. Image 1

## CRediT authorship contribution statement

**Tetsuo Yano:** Conceptualization, Methodology, Formal Analysis, Data Acquisition, Writing – Original Draft, Writing – Review & Editing. **Teppei Ito:** Investigation, Data Acquisition. **Yuya Kanehira:** Investigation, Data Acquisition. **Mei Yamada:** Investigation, Data Acquisition. **Hiromi Kimura-Suda:** Methodology, Formal Analysis, Writing – Review & Editing. **Hirotaka Wagatsuma:** Investigation, Data Acquisition. **Daisuke Inoue:** Conceptualization, Methodology, Writing – Review & Editing.

## Declaration of competing interest

The authors declare that they have no conflict of interest in this study.

HK has received research funding from 10.13039/100014421EA pharma (formerly known as Ajinomoto Pharmaceutical).

DI has received honoraria and research funding from 10.13039/501100004948Astellas Pharma, 10.13039/100002429Amgen, 10.13039/100014421EA Pharma, 10.13039/100010795Chugai Pharmaceutical, 10.13039/501100002336Daiichi Sankyo, Taisyo Pharmaceutical, and 10.13039/501100010486Teijin Pharma.
